# A New Radiological Classification for Massive Pulmonary Embolism (SPECS)

**DOI:** 10.1186/1749-8090-10-S1-A169

**Published:** 2015-12-16

**Authors:** Rachel JC Chubsey, Nicholas A Watson, Christopher MR Satur

**Affiliations:** 1 School of Medicine, David Weatherall building, Keele University, Staffordshire, ST5 5BG, UK; 2 Radiology Department UHNM NHS Trust, Royal Stoke University Hospital, Newcastle Road, Stoke-on-Trent, ST4 6QG, UK; 3 Cardiothoracic Surgery UHNM NHS Trust, Royal Stoke University Hospital, Newcastle Road, Stoke-on-Trent, ST4 6QG, UK

## Background/Introduction

Massive pulmonary embolism (PE) carries a mortality of 11% however current classifications do not identify those most at risk. Lack of direction prevents effective utilisation of options of treatment. We have undertaken this study to develop a radiological classification of massive PE that shows correlation with clinical outcome.

## Materials/Methods

The study was conducted using the radiology database to identify all patients who had a CT pulmonary angiogram during the 12 months of 2014. A total of 1923 CTPAs were conducted, of which 210 were reported as large PE. These were examined, those with a segmental PE or poor quality imaging were excluded, leaving 154, 91 (59%) females in the study. The classification assessed location of clot (L) in major pulmonary arteries (scores 1, 2 and 4), the degree of occlusion (o) (score 1,2 and 3) and the impact on the right ventricle (RVR) (scores 1,2 and 3). Interventricular septum morphology (S) was also assessed (scores 1, 2, 3 and 4). Multipliers were used obtain the total score, [(L × O) × RVR] + S. Maximum score 64. This score was correlated with clinical outcome.

## Results

Average age of 69 years (range 20-98 years), with 13 deaths, 4 primarily from PE (group A), the remainder from causes other than PE (group B). Group A had a median score of 28.5 (IQR 16.5-33.7) compared with survivors, median score of 6 (IQR 3-15), p = 0.005. 2 patients with scores 14 and 24 respectively died following thrombolysis and interventional radiology. 1 patient with a score of 33 survived after surgical pulmonary embolectomy.

## Discussion

The results from this study demonstrate that the UHNM PE score is able to distinguish those patients with a massive PE that are life-threatening. We suggest the score requires further evaluation.

## Conclusion

The UHNM PE Score, a new radiologic classification of massive PE that may be utilised in evaluating available therapies.

